# Biological tissue sample preparation for time-of-flight secondary ion mass spectrometry (ToF–SIMS) imaging

**DOI:** 10.1186/s40580-018-0157-y

**Published:** 2018-09-25

**Authors:** Sohee Yoon, Tae Geol Lee

**Affiliations:** Center for Nano-Bio Measurement, Korea Research Institute of Standards and Science (KRISS), Daejeon, 34113 Republic of Korea

**Keywords:** Sample preparation, ToF–SIMS imaging, Frozen hydrate analysis, Freeze drying, Adhesive tape mounting, Thaw mounting, Tissue section, Cholesterol, Analyte migration

## Abstract

Time-of-flight secondary ion mass spectrometry (ToF–SIMS) imaging is an analytical technique rapidly expanding in use in biological studies. This technique is based on high spatial resolution (50–100 nm), high surface sensitivity (1–2 nm top-layer), and statistical analytic power. In mass spectrometry imaging (MSI), sample preparation is a crucial step to maintaining the natural state of the biomolecules and providing accurate spatial information. However, a number of problems associated with temperature changes in tissue samples such as loss of original distribution due to undesired molecular migration during the sample preparation or reduced ionization efficiency make it difficult to accurately perform MSI. Although frozen hydrate analysis is the ideal sample preparation method to eliminate the effects of temperature, this approach is hindered by mechanical limitations. Alternatively, an adhesive-tape-supported mounting and freeze-drying preparation has been proposed. This paper provides a concise review of the sample preparation procedures, a review of current issues, and proposes efficacious solutions for ToF–SIMS imaging in biological research.

## Introduction

Mass spectrometry imaging (MSI) provides information on the spatial distribution and chemical composition of analyte molecules on complex surfaces by irradiating the sample with an ionized beam and recording the ion signal at different locations [1–3]. MSI enables label-free detection of biomolecules such as small organic molecules, metabolites, proteins, and DNA and can identify unknown molecules [1]. In addition, multivariable analysis can be used for simultaneous analysis of various compounds in numerous fields such as biotechnology, drug development, and medical research [1, 4].

The widespread adoption of MSI as an analytical tool for surface analysis depends on the successful resolution of one key issue: improvement in ionization efficiency of the conversion of target molecules into gas phase ions. Various methods have been developed to enhance this efficiency by analyzing the characteristics of the analyte [5–20]. Recently, atmospheric pressure ionization methods [5] were developed to generate the analyte ions in an atmospheric environment, followed by the introduction of the generated ions into a vacuum. However, various ionization methods in a vacuum have been previously investigated for high-resolution mass spectrometry imaging. Examples of MSI in a vacuum environment includes matrix-assisted laser desorption ionization (MALDI) MSI [6, 7], nanoparticle-assisted laser desorption ionization (LDI) MSI [8], surface-assisted laser desorption ionization (SALDI) MSI [9], time-of-flight secondary ion mass spectrometry (ToF–SIMS) imaging [10], and gas cluster ion beam (GCIB) ToF–SIMS imaging [11, 12]. Each method utilizes UV-activated organic molecules [6], organic metal molecules [13], nanoparticles [14–16], nanowires [9, 17], nanostructured surfaces [18–20], etc. to enhance the ionization of biomolecules on the surface of a sample, or a gas-cluster ion beam (GCIB) [11] as the ionization source.

Preparing the tissue slices in order to preserve the original location and chemical information of the analytes in the sample is another important step in biological MSI [1–3]. The sample preparation procedure involves a series of steps that include sample collection, storage, sectioning, drying, and sample plate configuration. In the case of biological samples, there are a number of factors to consider such as the moisture content, the robustness of the tissue, and whether the sample is fixed [1, 21–25].

Given the importance of sample preparation, ToF–SIMS is advantageous in that samples can be analyzed directly without applying additional organic molecules to the tissue to facilitate ionization, and enable label-free detection [26–28]. In addition, this technology is emerging as a biological MSI because ToF–SIMS imaging also achieves high spatial resolution (50–100 nm) using focused ion beams [29–32]. This review presents a detailed overview of tissue sample preparation, in addition to current issues and solutions for bio-ToF–SIMS imaging.

## ToF–SIMS imaging

ToF–SIMS is a surface mass spectrometry method in which a sample is directly irradiated with an accelerated ion beam, and the generated secondary ions are measured using a time-of-flight analyzer [33]. This method has been widely used as a measurement technique in related industries, such as depth profiling of semiconductor thin films made of inorganic material [34–36], because it can analyze a molecular layer at several nanometers below the sample surface with sub-micrometer lateral resolution [37–40]. However, the technique is limited to intact bio-molecule analysis and MSI application because of the low production yield of secondary ions and the extensive fragmentation of molecular ions due to the high energy of the ion beam [41, 42]. The introduction of metal cluster ion beams such as Au_3_^+^ [43] and Bi_3_^+^ [44] enables the analysis of biomolecules with low molecular weight such as metabolites and lipids [30, 41, 45, 46]. C_60_ [42, 47] and Ar cluster ion beams [48, 49] enable the measurement of molecular ions with high molecular weight such as peptides and proteins from biological samples [12, 50–52]. In addition, fragmentation is reduced, and the molecular ion signal is increased. In other words, gas cluster ion beams (GCIB) dramatically increase the applicability of ToF–SIMS imaging to biological analysis.

ToF–SIMS with GCIB has been widely used to study in vivo mechanisms and discover disease biomarkers by facilitating mass spectrometry images of various living tissues including the brain [52–57], eye [58–60], liver [61], kidney [62, 63], skin [64–66], stomach [67], etc. In particular, in studies of brain tissues [54, 68, 69], where the structures are complex and play an important role in each region, MSI is advantageous because it can assist in the determination of the spatial distribution and quantification of specific biomolecules.

The first step in ToF–SIMS imaging is to prepare the biological tissue. For this reason, it is important to understand the physicochemical properties of the tissues and target molecules since they are typically analyzed using the MSI approach. Despite sample diversity, several sample preparation methods have been well-established for MSI through a process of trial and error [1, 3, 22, 25]. A few of these procedures are presented in the following sections.

## Sample preparation for ToF–SIMS imaging

**Fig. 1:**
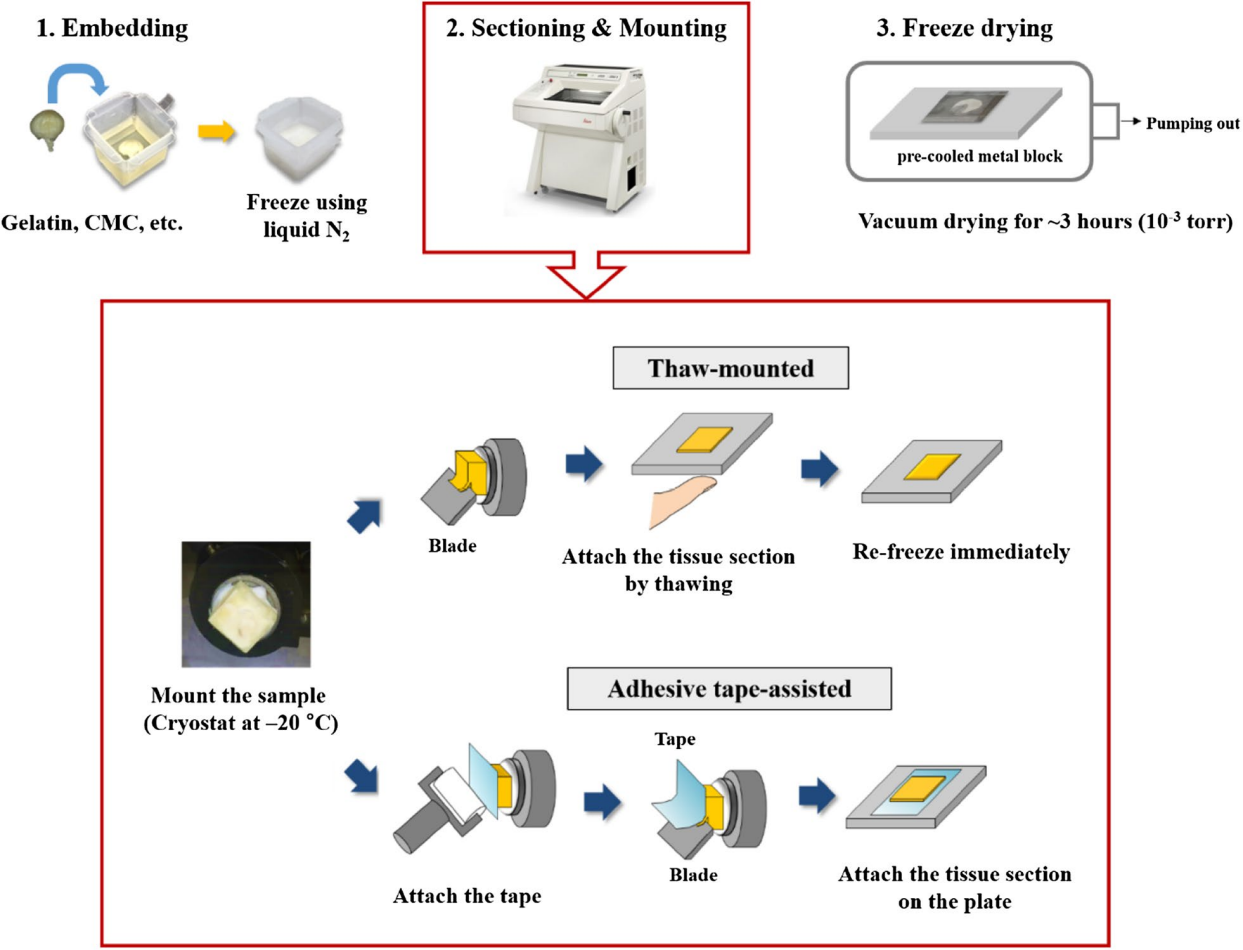
Typical work flow of tissue section preparation for MSI (Reproduced from ref. [85] with permission, © Springer)

Preparation of tissue samples generally involves fixing cryopreserved biological samples, sectioning them with a thin blade, mounting onto the sample plate, drying, followed by the analysis procedure as shown in Fig. 1. Great care should be taken in the process of fixing, and determining section thickness and mounting type, as well as the process of drying, since these parameters can change according to the sample species, their size, the degree of moisture content, firmness, etc. This section will review the preparation of tissue samples for ToF–SIMS imaging.

### Sectioning

MSI can be most effectively performed when the surface of the sample to be irradiated with the ionization beam is flat or evenly uniform, hence necessitating the sectioning of the tissue samples (ex., biopsy samples) into thin slices. Tissue samples frozen at cryogenic temperatures can be immediately sectioned and prepared for mounting after the embedding process. If the sample is small, fragile, or if there is a solid cell wall such as in the case of a plant sample, or if the intracellular space inside the sample is large and filled with water, the sample should be embedded with specific materials [1, 22]; in general, the embedding materials used in MSI are optimum temperature cutting (OCT) [70], carboxymethyl cellulose (CMC) [71], gelatin [72, 73], or ice [74]. OCT compounds are mixtures of polyethylene glycol and can diffuse into tissues and smear across the surface during sectioning [70, 75]. For this reason, care should be exercised in the choice of compounds prior to sectioning in MSI. CMC and gelatin compounds are more suitable to MSI than OCT [25, 70]. While CMC is generally selected as the embedding material in relative large tissues, gelatin compounds are used mainly for small tissue samples [22].

A common method to section frozen tissue samples is via cryosectioning using a microtome. The frozen or frozen embedded tissue sample is placed in a chamber set at approximately − 20 °C and then subsequently sliced to an optimal thickness using a pre-cooled blade. Because the sectioning process is performed at a low temperature, the metabolic process in the tissue can be quenched, and the tissue sectioning can be rapidly performed. In MSI, animal tissue sections are generally cut to a thickness of 10–20 μm [75].

### Mounting

The process of fixing the sectioned tissue to the sample plate is called mounting. The most commonly used method for mounting biological samples is the thaw-mounted method. In this method, a thin frozen section of several tens of micrometers is placed on a precooled (− 20 °C) metal plate or indium-tin-oxide (ITO) glass. Then, the tissue is fixed using heat from a finger as shown in Fig. 1. This approach is mainly used to attach brain and organ tissues of animal samples.

Because plant samples are relatively large, fragile, and less adhesive because of their low lipid content compared to animal samples, it is difficult to immobilize them onto the sample plate using the thaw-mounted method [2, 76, 77]. Alternatively, then, a tissue sample can be mounted using an adhesive tape. Kawamoto’s group [76] performed MSI on the frozen section of an entire rat by attaching fragile samples using an adhesive tape assisted method. The CryoJane Tape-Transfer System^®^ [77] also uses an adhesive tape to attach a frozen sample block and transfer it to a pre-coated glass slide. After UV treatment to remove the CryoJane tape, only the sample section remains on the slide. These approaches can be used to prepare animal samples as well as tissue sections of plant specimens.

### Drying

Fresh tissue samples may shrink in a vacuum environment, and biological processes may still be activated, leading to degradation of the tissue samples during MSI analysis [78]. The mounted tissue sample should be dried prior to ToF–SIMS analysis, which is typically performed at high vacuum levels of 10^−9^ to 10^−10^ Torr. In general, the drying method used is freeze-drying [43, 54, 79]. The mounted tissue sample is immediately introduced into a vacuum chamber, which is slowly pumped until the temperature of the sample reaches room temperature while the water content is removed from the tissue. When the mounted sample is placed in the vacuum chamber, it may be placed on the pre-cooled metal frame, or the chamber is maintained at a low temperature to allow the temperature of the tissue sample to change as slowly as possible. The chamber is pumped out at 10^−3^ Torr for 1–3 h to facilitate drying.

Other drying methods include air drying under nitrogen purging conditions [80] or dehydration [81] through solvent washing for further treatment of the tissue surface, followed by drying at room temperature. However, freeze-drying, air-drying, and room-temperature drying are used to produce dry samples in preparation for ToF–SIMS imaging since these techniques can be performed without applying additional organic compounds for ionization.

## Sampling issue in ToF–SIMS imaging

The critical importance of sample preparation in MSI has been highlighted in the previous sections. Analyte relocation is a possibility and occurs depending on the mounting type of the sectioned tissue, the drying process of the mounted sample, and the temperature of the sample during ToF–SIMS MSI. This section will review some of the prevalent issues associated with each sample preparation step.

### Sample temperature during mounting and drying

As previously mentioned, there are two ways to mount sliced tissue samples. One is by thaw-mounting, and the other is via an adhesive tape-supported method as shown in Fig. 1. The main difference between the two methods is the application of heat to the tissue sample during mounting in the former.

Sjövall et al. [79] observed changes in the detection position and signal intensity of cholesterol in rat brain tissue when the temperature of the tissue prepared using the thaw-mounted method was changed from 130 to 50 °C under vacuum. As a result, the signal from the cholesterol was strong above 0 °C, and the signal pattern was found to be the same as that recorded at a high temperature. This was the case even though the temperature of the brain tissue was increased to a high value then lowered again to below 0 °C. It is unclear why cholesterol migrates to the tissue surface. However, it has been reported that a matrix effect of cholesterol suppresses detection of other analytes when the cholesterol is diffused and extensively distributed over the tissue surface during sample preparation. Bich et al. [10] performed a ToF–SIMS depth profile analysis of lipids in rat brain tissue using dual beam ionization including an Ar_4000_^+^ cluster ion beam for sputtering, and a Bi_3_^+^ ion beam for analyzing. When the ratio of each beam was optimized for the increase of the signal intensities, the depth profile of lipids such as fatty acid (FA), sulfatide, and phosphatidylcholine (PC) was constant throughout the tissue section. However, a 3D image revealed that cholesterol was distributed only at the tissue surface. Debois et al. [82] performed a depth profile of rat brain tissue using a ToF–SIMS equipped with a C_60_^+^ cluster ion gun and found that almost all the lipids in the tissue were concentrated at 2–300 nm from the surface, with inconsistent distribution. This means that not only cholesterol, but also other biomolecules such as lipids likely migrate in the tissue due to changes in the sample temperature.

**Fig. 2:**
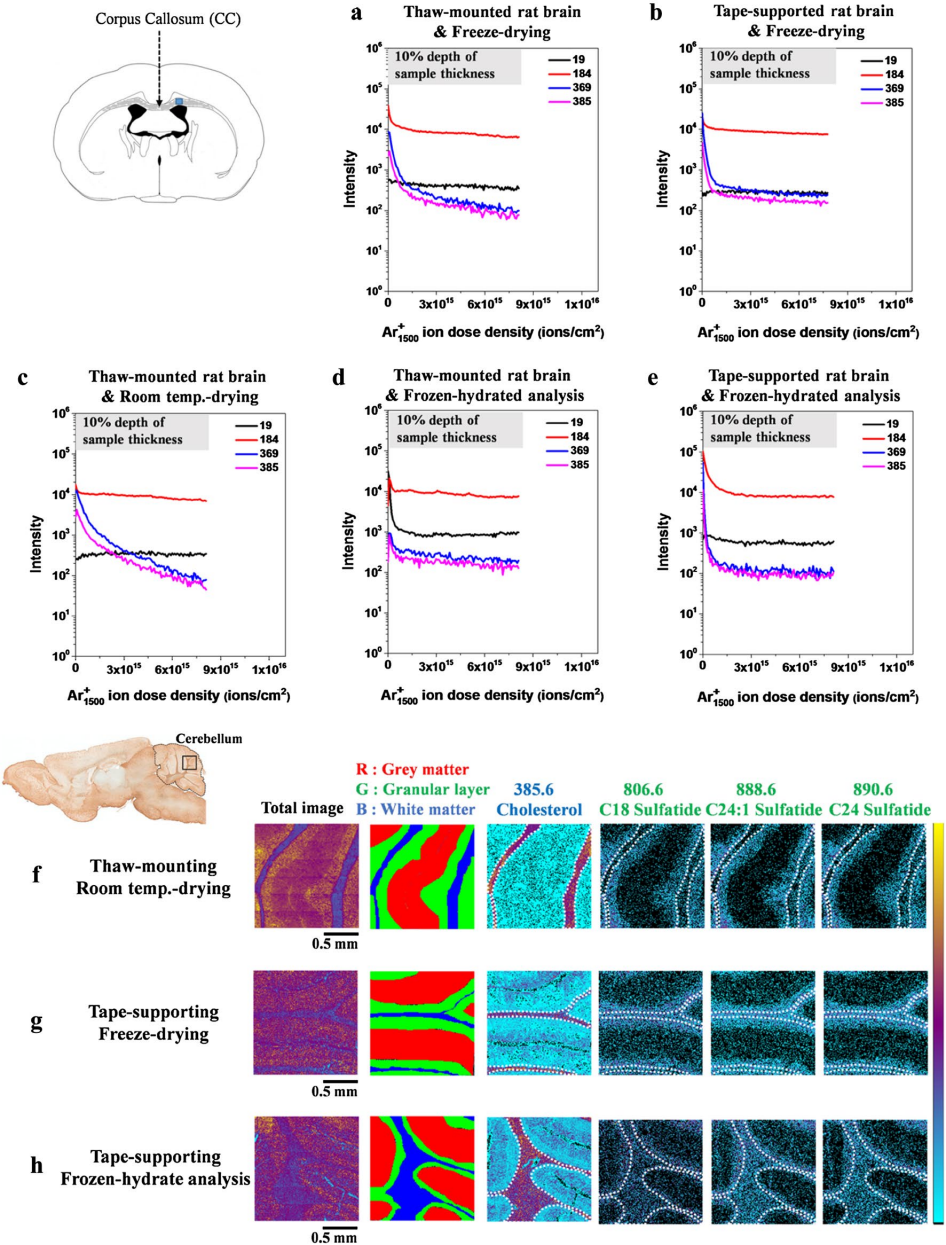
ToF–SIMS depth profiles of H_3_O^+^, C_5_H_15_NPO_4_^+^, C_27_H_45_^+^, and C_27_H_45_O^+^ peaks in corpus callosum in coronal section and ToF–SIMS images of cholesterol and sulfatide ion peaks of the rat brain tissue. The blue square indicates the position for depth profile acquisition. Depth profiles of H_3_O^+^ at *m/z* 19, C_5_H_15_NPO_4_^+^ at *m/z* 184, C_27_H_45_^+^ at *m/z* 369 and C_27_H_45_O^+^ at *m/z* 385 obtained at the corpus callosum prepared using **a** thaw-mounted rat brain on a stainless steel plate and freeze-drying, **b** tape-supported rat brain on a stainless steel plate and freeze-drying, **c** thaw-mounted rat brain on a stainless steel plate and room temperature-drying, **d** thaw-mounted rat brain on a stainless steel plate and frozen-hydrate analysis, and **e** tape-supported rat brain and frozen-hydrate analysis. Two ions, *m/z* 369 (C_27_H_45_^+^) and *m/z* 385 (C_27_H_45_O^+^), are the characteristic peaks of cholesterol, known as [M+H–H_2_O]^+^ and [M−H]^+^, respectively. In ToF–SIMS imaging, the rat brain was cut in the sagittal direction. Negative ion images obtained at the cerebellum region prepared by using **f** thaw-mounted rat brain on a stainless steel plate and room temperature-drying, **g** tape-supported rat brain and freeze-drying, and **h** tape-supported rat brain and frozen-hydrated analysis. Scale bar is 0.5 mm. MC stands for maximal counts (Reproduced from ref. [83] with permission, © John Wiley & Sons, Ltd)

The difference in the cholesterol distribution of the two mounting methods was recently investigated by Lee’s group [83–85]. Shon et al. [83] compared the results of the ToF–SIMS depth profiles obtained by freeze-drying rat brain tissue using the thaw-mounted and adhesive tape-supported methods. The results are shown in Fig. 2a, b. In the adhesive tape-supported method, there was little observable change in the amount of cholesterol in the depth direction as shown in Fig. 2b. However, in the case of the thaw-mounted method, the amount of cholesterol decreased from the surface of the brain tissue toward the sample plate (bottom of the tissue section) as shown in Fig. 2a.

**Fig. 3:**
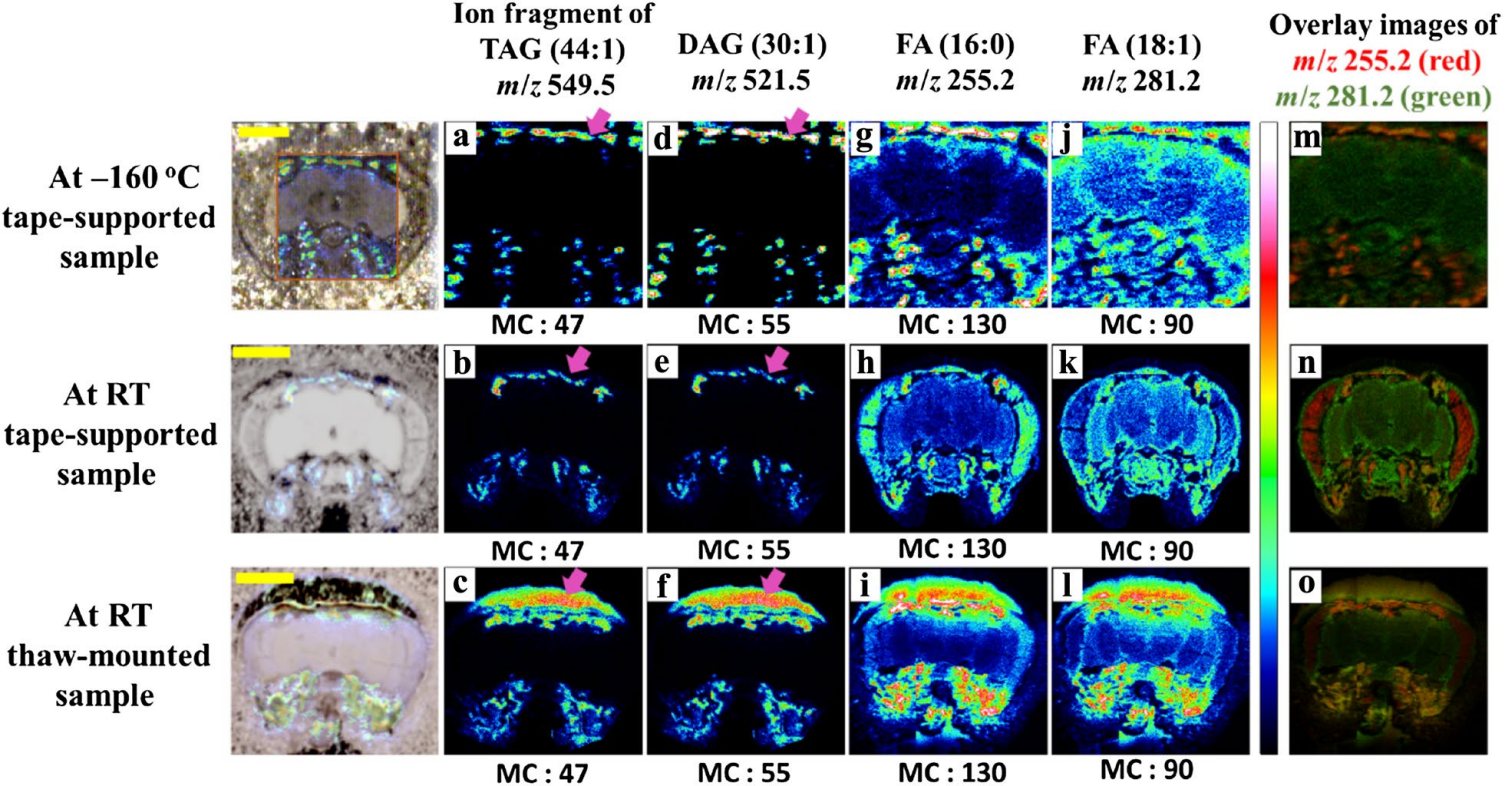
Comparison of *Drosophila* brains prepared by three different preparation and analysis procedures. Tape-supported at low temperature (**a**, **d**, **g**, **j**, **m**), at room temperature (**b**, **e**, **h**, **k**, **n**), and thaw-mounted at room temperature (**c**, **f**, **i**, **l**, **o**). The distributions of positive ions at *m*/*z* 549.5—ion fragment of TAG (44:1), and 521.5—DAG (30:1), respectively, and negative ions at *m*/*z* 281.2 [FA (18:1), oleic acid] and *m*/*z* 255.2 [FA (16:0), palmitic acid]. Scale bar is 200 μm. MC stands for maximal counts (Reproduced from ref. [84] with permission, © John Wiley & Sons, Ltd)

**Fig. 4:**
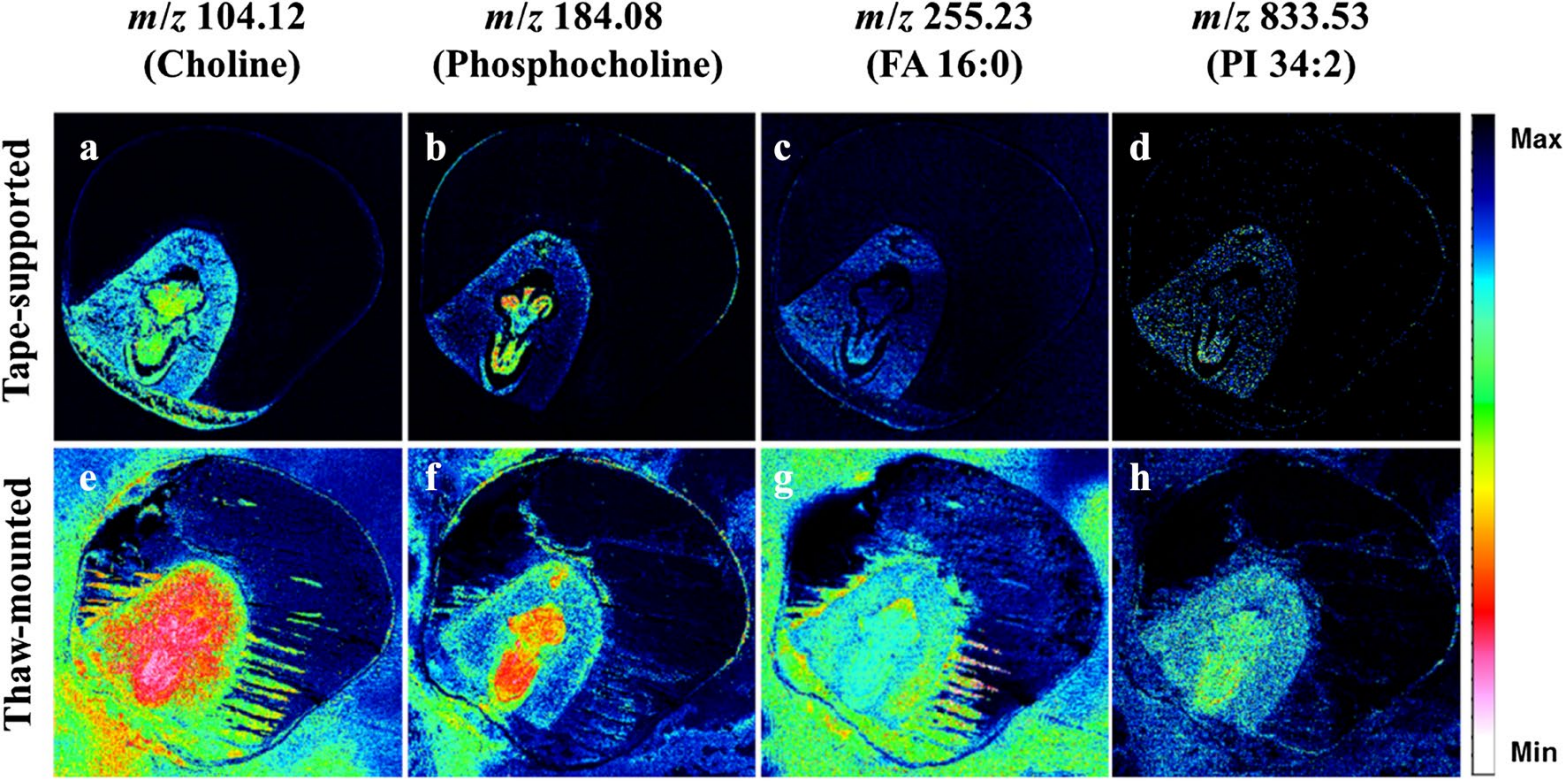
ToF–SIMS images of corn seed tissue prepared by the adhesive tape-supported and thaw-mounted methods. Molecular distribution of **a**, **e** choline, **b**, **f** phosphocholine, **c**, **g** palmitic acid, and **d**, **h** phosphatidylinositol (PI) 34:2 (Reproduced from ref. [85] with permission, © Springer)

Analyte relocation has also been observed in samples other than rat brain tissue. Uyen et al. [84] performed ToF–SIMS imaging of *Drosophila* head sections that were prepared by the two mounting methods and freeze-dried using the same method. In order to prevent charge accumulation during analysis, a conductive double-sided carbon tape was used for mounting. They compared the distributions of diacylglycerol (DAG), triacylglycerol (TAG), and FA molecules which are known to be sensitive to changes in sample temperature in the mass spectrometric images of brain samples prepared by the two mounting methods as shown in Fig. 3. The size of the head tissue was found to be reduced by about 30% in the thaw-mounted preparation compared to the adhesive tape-supported method. According to the authors, this was due to a process caused by moisture accumulation on the tissue surfaces finger heat was applied. Moisture accumulated on the tissue and a phase change to ice occurred during re-freezing, which caused cell wall breakage due to volume expansion. Water was subsequently released between the collapsed cell walls, and a wrinkled tissue sample of a reduced size was produced. In the ToF–SIMS imaging, TAGs, the main components of the head fat body, were measured via strong signals outside the tissue using the thaw-mounted method (as in Fig. 3c). In the case of the adhesive tape-supported method, the spatial distribution of the TAGs was well conserved (as in Fig. 3a, b). In addition, ToF–SIMS images revealed that the spatial information of the DAGs (as in Fig. 3d, e) and the palmitic acid molecules (as in Fig. 3g, h) was well maintained for the adhesive tape-supported preparation. However, this distribution was observed outside the tissue or at other locations in the tissue in the case of the thaw-mounted preparation (as in Fig. 3c, f, i). Kim et al. [85] demonstrated that the image distortion in corn seed tissues was caused by the temperature change of the tissue sample that occurred during the mounting and drying process. Corn seeds contain more than 90% of the constituents of the endosperm of starch or cellulose [2], so, unlike the embryo part with many lipid components, the endosperm is less adhesive and is difficult to adhere to the sample plate in the thaw-mounted method. As a result, numerous cracks were produced. However, the adhesive tape was able to adhere uniformly without any breakage of the sample regardless of the degree of adhesion as shown in Fig. 4. Just as in the case of the *Drosophila* head tissue, the size of the corn seed tissue section mounted by thawing was reduced compared to that prepared using the adhesive tape; the structure inside the embryo was also distorted. Based on the ToF–SIMS image of the corn seed tissue section mounted by the adhesive tape, choline, phosphatidylcholine, palmitic acid, and phosphatidylinositol molecules were clearly distinguishable and were observed without loss of location information as shown in Fig. 4. On the other hand, ToF–SIMS images with accurate location information could not be obtained due to the relocation of lipid molecules in the corn seed tissue sample mounted without adhesive tape.

### Measurement temperature

The ideal measurement method for ToF–SIMS imaging is via frozen hydrate analysis [86, 87] which measures the sample at a cryogenic temperature of − 160 °C. As mentioned earlier, the temperature change of the sample affects the results of the imaging analysis. Therefore, ToF–SIMS imaging via frozen hydrate analysis can eliminate the possibility of redistribution of analytes in tissue samples due to temperature changes during the measurements or drying process.

Phan et al. [88] performed ToF–SIMS imaging of *Drosophila* head tissue sections at cryogenic and room temperatures, and compared the differences in the results according to the sample temperature. The molecular distribution of DAGs was detected in the freeze-dried whole *Drosophila* brain tissue, but the frozen hydrate analysis showed that the DAGs signal was detected only in the clear domains of the brain tissue. DAGs can be generated by fragmentation of TAGs or by hydrolysis of phospholipids. In the results of the frozen hydrate analysis, the authors found that the PC head group and DAGs were distributed in different regions, indicating that PC containing phospholipids did not originate from DAGs. Given that DAGs molecules were detected throughout the brain tissue when the measurement was performed at room temperature, frozen hydrate analysis can provide an accurate mechanism for the generation of DAGs, which are messenger molecules that are important for cell growth and function determination.

The effect of sample temperature during ToF–SIMS measurement and freeze-drying process also appears in the results of rat brain tissue imaging (Shon et al. [83]). The results shown in Fig. 2 indicate the difference in the depth profile of cholesterol when the rat brain tissue was prepared using the thaw-mounted and freeze-dried approaches, measured at room temperature and − 160 °C. In the frozen hydrate analysis as in Fig. 2d and e, the cholesterol signal intensity did not change even when the Ar_1500_^+^ ion dose for sputtering was increased, but the intensity rapidly decreased when the ion dose was increased when measured at room temperature, as shown in Fig. 2c. However, when the rat brain tissue was mounted and prepared using the adhesive tape-supported method and freeze-drying, and measured at room temperature, the cholesterol ion signal intensity was observed to be almost constant regardless of any increase in the Ar_1500_^+^ ion dose intensity, as shown in Fig. 2b. The thermal conductivity difference between the polymer with a low thermal conductivity, which is normally used in the tape and the metal plate with a high thermal conductivity, prevents the occurrence of heat transfer to the tissue sample during the freeze-drying process. According to the authors, this thermal conductivity difference prevents cholesterol migration during freeze-drying when the tissue is mounted with adhesive tape, so there is no difference in the ion signal intensity between the measurement temperatures as shown in Fig. 2b and e. ToF–SIMS imaging results by Uyen et al. [84] also support this explanation. Positive ion mass spectral images of a *Drosophila* head section prepared by the adhesive tape-supported and freeze-dried methods were observed to be very similar to the images obtained from frozen hydrate analysis as shown in Fig. 3.

## Optimization of sample preparation for ToF–SIMS imaging

During sample preparation for ToF–SIMS imaging, the temperature of the tissue sample can cause analyte relocation which results in a mass spectrometric image that contains distorted spatial information. This section describes current methods that address this issue and discusses techniques to improve the analyte ion signal intensity.

### Enhancement of ionization efficiency—TFA and ammonia treatments

Ion signal intensity is the most critical factor in ToF–SIMS image quality and is dependent on the ionization efficiency of the analytes. To increase ionization efficiency, additional aiding compounds are applied to the tissue sample [89, 90], or GCIB [11, 12, 50–52, 82–84] may be used to enhance the survival yield of the molecular ions with high molecular weights or fragile biological polymers.

In matrix enhanced-SIMS (ME-SIMS) [89], the ionization efficiency can be improved by providing a proton source from the organic compounds used as a matrix in MALDI. However, this method is disadvantaged by the occurrence of analyte rearrangement when the solvent is sprayed into the tissue sample as the matrix is applied. As a result, spatial resolution is often reduced, due to the larger size of the matrix crystals compared to the primary ion beam size. Metal-assisted SIMS (MA-SIMS) [90] which utilizes metal molecules rather than organic matrices, are hindered by the problem of selective ionization of analytes since the adduct formation of metal cation with specific analyte can increase the ionization efficiency of this specific analyte.

A primary ion beam with high energy easily causes fragmentation of molecular ions, which makes it difficult to detect the intact ions. In ToF–SIMS, the detection probability of intact molecular ions can be increased by introducing a metal cluster ion beam or a GCIB, which is widely used for large biomolecule detection [12, 50–52, 82–84]. However, undesired distribution of specific molecules on the sample surface adds difficulty to the image analysis process even when cluster ion beams are applied to ToF–SIMS, due to its high surface sensitivity (1–2 nm top-layer). In particular, cholesterol or vitamin E easily migrate to the tissue surface and suppress ionization of other analytes if the temperature is not maintained at cryogenic temperatures during sample preparation [53, 79, 83, 86, 87, 91].

Angerer et al. [53] observed that the signal intensity of sulfatides and ceramides among the various lipid molecules was extremely strong when ammonia treatment was applied to rat brain tissue. When the ammonia-treated and frozen-hydrated tissue samples were compared, it was observed that the signal patterns of two lipid molecules in the ammonia-treated tissue were almost similar to those of the frozen-hydrated tissue. After ammonia treatment, cholesterol crystals on the tissue surface were still visible, but were mostly reduced to the frozen hydrate level. The authors explained that sulfatides in the lower parts of the tissue were detected because the physically coated cholesterol on the sample surface was successfully removed.

**Fig. 5:**
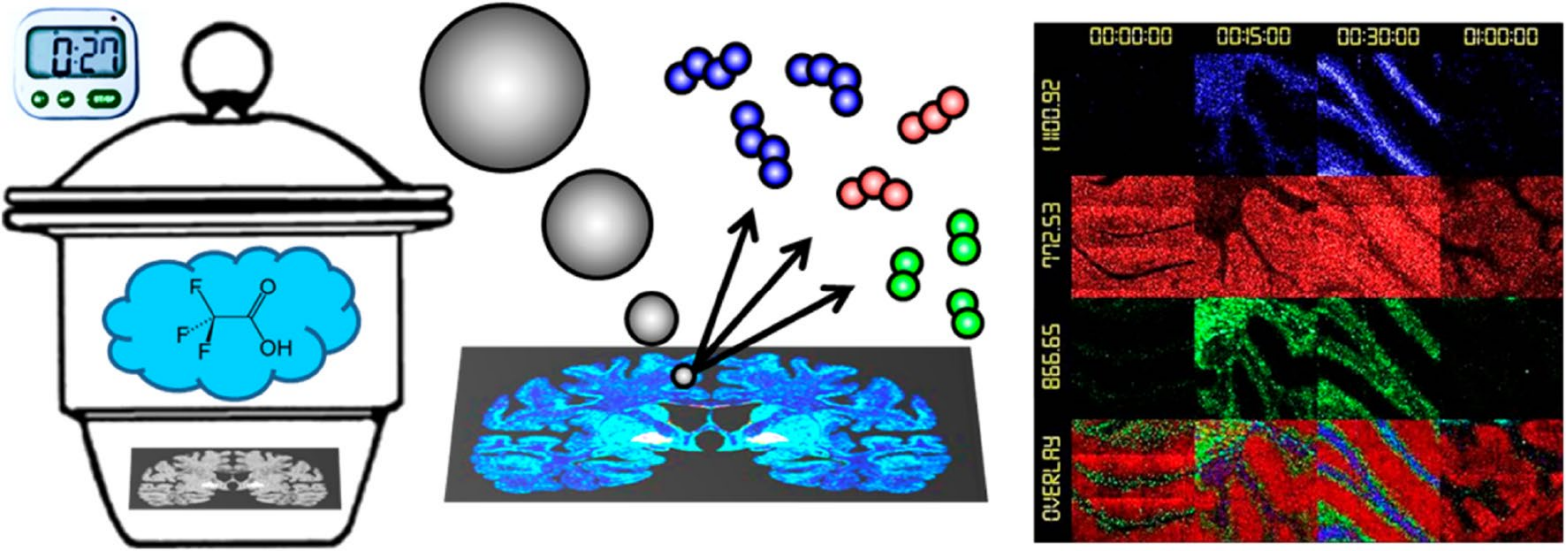
Schematic of the workflow for lipid imaging with SIMS following exposure to TFA vapor. Tissue slices on microscope slides with an electrically conductive coating (indium tin oxide, ITO) were placed in a desiccator containing 300 μL of TFA for different lengths of time. The samples were transferred to the mass spectrometer for SIMS analysis where the tissue was bombarded with a focused beam of Ar_4000_^+^ ions. Several hundred peaks with distinct mass signals were detected of which selected lipid species were imaged (Reproduced from ref. [91] with permission, © American Chemical Society)

Another study by the authors suggested a method for improving signal intensity by exposing the sample to trifluoroacetic acid (TFA) vapor [53, 91] as shown in Fig. 5. TFA vapor exposure to rat brain tissue removed cholesterol coated on the sample surface, thus enhancing the ionization efficiency and increasing the ion intensity of other analyte molecules. Exposure to TFA vapor caused only the cholesterol signal intensity to be reduced, whereas the signal intensity of other phospholipid molecules increased. Moreover, signals from large molecules with high molecular weight which are not often seen in conventional measurements, could be measured. In particular, the enhancement of the analyte ion signal intensity was effective in the positive ion mode where cholesterol ion is formed. According to the authors, the effect of the TFA vapor exposure is to remove cholesterol from the tissue surface by forming a more volatile species due to the reaction between TFA and cholesterol.

### Frozen-hydrated analysis—signal enhancements

The frozen hydrate sample preparation is an optimal method to perform the analysis while maintaining the sample hydrate state and the material distribution in the sample in a high vacuum as close as possible to the native state [53, 86–88]. Typically, the sectioned tissue sample is mounted by thawing on a metal plate or ITO glass, followed by direct fixing to the sample plate holder which is cooled with liquid nitrogen or cooling gas and evacuated for several hours to prepare a high vacuum. The analysis is performed in a chamber filled with argon gas to prevent water condensation on the surface of the sample [25, 53, 87]. During measurements of the prepared frozen hydrated tissue samples, the temperature should be maintained at a cryogenic temperature of − 160 °C.

Frozen hydrate analysis has the effect of improving the analyte ion signal intensity in addition to achieving the closest native state of the sample during imaging [53, 82, 83, 86–88, 91]. Under this frozen hydrate environment, the signal intensity increase from small peptides, drugs, and lipids was proved by surface analysis and depth profiling using ToF–SIMS [42, 53, 82, 83, 86–88, 91–93]. Changes in the sample temperature leads to the possibility that molecules with high mobility migrate to the tissue surface and form a physical surface layer, thereby reducing the chance that other molecules will come into contact with a proton. If the temperature is kept at a cryogenic temperature, there is no molecular movement, and the ionization of the analyte is undisturbed, so the signal intensity is relatively improved. One of the most important points in the frozen hydrate analysis process is to avoid water condensation on the sample surface. Piwowar et al. [94] prepared HeLa cells in a frozen hydrated state using liquid ethane and measured the specific lipids via C_60_^+^ ToF–SIMS imaging. The authors reported that liquid ethane has the effect of preventing water condensation on the sample surface in a high vacuum.

Because frozen hydrate analysis provides undistorted molecular distribution information, frozen hydrated ToF–SIMS imaging enables drug-related biological studies such as drug-induced changes in biomolecules and the monitoring of drug metabolism [95, 96]. A study from Ewing and Sjövall’s group [88, 95, 96] demonstrated the spatial distribution of phospholipids in the brain and DAGs, using statistical analysis based on ToF–SIMS images of a *Drosophila* head section prepared under frozen hydrated conditions. The authors set up a ToF–SIMS imaging method for *Drosophila* brain analysis that could facilitate the investigation of the relationship between drug efficiency and brain function by comparing the lipid profiles of brain tissue treated with the drug to a normal brain [88]. Phan et al. [95] investigated the phospholipid species affected by the drugs, by monitoring the changes to the molecular distribution between non-treatment and tissue treated with methylphenidate, a prescription drug for attention-deficit/hyperactivity disorder (ADHD). Philipsen et al. [96] performed ToF–SIMS frozen hydrate analysis on the changes in lipid distribution due to cocaine and methylphenidate in the nervous system. Significant changes in phospholipids were observed in the central region of the fly bran treated with the two drugs, but it was discovered that they each had opposite effects on the brain lipid structure.

### Tape-supported mounting and freeze drying

Frozen hydrate analysis is an ideal way to perform ToF–SIMS imaging of bio-tissue samples in the absence of chemical information and positional information distortion. However, the measurement range is limited, and the formation of water clusters can make spectral analysis difficult. In addition, there is a mechanical limitation on the stage scan for large area, typically larger than 500 μm × 500 μm, because measurements in frozen hydrate analysis, are performed at cryogenic temperatures. ToF–SIMS imaging analysis at room temperature using adhesive tape has been attempted to achieve results that are comparable to those obtained using frozen hydrate analysis.

Several studies [76, 77, 97, 98] have reported on mounting tissues using adhesive tape, particularly in cases where it is difficult to fix the sample plate in a thaw-mounted manner in MALDI MSI due to size. Attaching fragile tissues using adhesive tape [76] and the CryoJane Tape-Transfer System^®^ [77] can aid in preparing animal samples as well as tissue sections of plant samples. Lee’s group is actively conducting metabolite studies with MALDI-MS of maize seed section [97] and lipid mapping in a single cell of zebrafish embryo [98] by utilizing CryoJane tape in sample preparation. However, Kim et al. [85] recently reported on ToF–SIMS imaging results using adhesive tape. They compared ToF–SIMS images of corn seed tissues acquired using thaw-mounted preparation and 3 M tape mounted method. Unlike the thaw-mounted method, MSI results with undistorted location information of fatty acids and phospholipids present in the corn seed tissue were acquired when the corn seed tissue was mounted using adhesive tape. This was achieved without contamination of the embedding substance gelatin. A follow-up study by Lee’s group [83] described the polymer material in the adhesive tape as acting like insulation between the sample plate and the tissue sample, preventing direct thermal energy from being applied to the sample. These results show that ToF–SIMS imaging of hard plant tissue samples is possible without image distortion and with reduced adhesion properties when the appropriate sample preparation procedure is utilized.

Metzner et al. [99] measured toxic elements by ToF–SIMS imaging to trace the nutrient transport pathway of plants at the cellular and tissue level. MSI results for Na, K, and Rb, in addition to cryo-SEM images of a French bean sample prepared via frozen hydrate, were obtained. The subcellular details of the tissue structure were well preserved during the analysis process. It was suggested by the authors that frozen hydrate ToF–SIMS imaging could provide quantitative information on plant nutrition.

Uyen et al. [84] compared ToF–SIMS imaging with that of frozen hydrate analysis results based on the difference in the sample preparation method of a *Drosophila* head section (Fig. 3). The authors suggested that the spatial distribution of analytes in the tissue attached with adhesive tape was almost the same as the result for the frozen hydrate analysis. This was the case even without additional thermal energy including finger heat and even if the tissue was dried slowly, that is, excellent freeze-drying in which the section is placed on the pre-cooled stainless steel plate kept at − 80 °C.

A study by Shon et al. [83] arrived at the same conclusion. The study used depth profiling and MSI to show that adhesive tape-supported mounting and strict freeze-drying preparation of rat brain tissues facilitated ToF–SIMS imaging without the undesired migration of analytes. As shown in Fig. 2f–h, the results of the frozen hydrate analysis of the samples prepared by the two mounting methods (thaw-mounting or tape-supported mounting) revealed that cholesterol was uniformly distributed throughout the tissue with regard to depth, regardless of the mounting procedure as shown in Fig. 2d, e. Therefore, they suggested that finger heat was not a major cause of cholesterol migration, although there was to some extent cholesterol migration in the case of thaw-mounting. They also showed that cholesterol migration was induced to a greater extent in the drying process by comparing freeze-drying to room temperature-drying under strict temperature control conditions as shown in Fig. 2c. The spatial distribution of cholesterol and sulfatide in ToF–SIMS images of the cerebellum region of the rat brain at cryogenic temperature as in Fig. 2h was the same as that of the tape-supported and freeze-dried rat brain shown in Fig. 2g. Based on the results, it was suggested that accurate MSI can be performed at room temperature when the sample is prepared by freeze-drying under temperature control subsequent to adhesive tape-supported mounting of biological tissue.

## Conclusion

MSI has become increasingly important in the field of biotechnology research where spatial information in tissue samples is important. Examples of these include biomarker discovery for degenerative brain disease [100–102] and cancers [103], and drug tracking in the body [104]. Based on the high spatial resolution, sample surface sensitivity, and statistical analysis, the application of ToF–SIMS imaging to biological studies is on the rise. This review summarizes the preparation procedures of tissue samples to determine the accuracy of ToF–SIMS imaging, recent trends in the field, and discusses different approaches for improving or resolving issues associated with the technique. Notably, in the sample preparation process, unexpected analyte relocation causes distorted of MSI results. Recently, the suppression effect that occurs when an excessive amount of cholesterol migrates to the tissue surface and interferes with measurements of other substances has been highlighted as a serious problem in ToF–SIMS imaging analysis of brain tissue sections. To date, frozen hydrate analysis is the best method to eliminate the problems related to sample preparation and to perform accurate MSI. From sample preparation to measurement, the sample temperature is kept at a low value between − 160 and − 80 °C, so that the analysis can be performed in a state that is closest to the natural state of the sample. If a large area sample analysis is required, the tissue samples can be prepared using the adhesive tape-supported method and freeze-drying to obtain nearly similar imaging results to frozen hydrate analysis. ToF–SIMS bio-imaging with accurate molecular distribution information can then be performed because the tissue samples can be prepared without the migration of analytes including cholesterol. This is performed through the by controlling the mounting and the drying process of the tissue without the application of additional thermal energy.

ToF–SIMS bio-imaging can be complemented with high-resolution optical imaging such as cryo-SEM [98, 105] or other surface analysis imaging techniques [106, 107] to perform biological sample analysis on specimens of various sizes and types. It is expected that improvements in the characteristics of these systems such as the spot size of ion beam, selection of cluster ion beam type, and selectivity of depth direction for sample analysis together with the optimal sampling method, will further enhance the bio-field expansion of ToF–SIMS imaging.
